# A Tutorial on Net Benefit Regression for Real-World Cost-Effectiveness Analysis Using Censored Data from Randomized or Observational Studies

**DOI:** 10.1177/0272989X241230071

**Published:** 2024-02-12

**Authors:** Shuai Chen, Heejung Bang, Jeffrey S. Hoch

**Affiliations:** Division of Biostatistics, Department of Public Health Sciences, University of California, Davis, CA, USA; Center for Healthcare Policy and Research, University of California, Davis, Sacramento, CA, USA; Division of Biostatistics, Department of Public Health Sciences, University of California, Davis, CA, USA; Center for Healthcare Policy and Research, University of California, Davis, Sacramento, CA, USA; Division of Health Policy and Management, Department of Public Health Sciences, University of California, Davis, Sacramento, CA, USA; Center for Healthcare Policy and Research, University of California, Davis, Sacramento, CA, USA

**Keywords:** censoring, cost-effectiveness analysis, net benefit regression, non-randomized study, observational data, propensity scores

## Abstract

Given the increasing popularity of person-level cost-effectiveness analysis using “real-world” data, there is a clear need to understand and use methods for observational data. When the cost-effectiveness data are subject to censoring, ignoring censoring is especially error prone for heavily censored data. We summarize best practice and provide a hands-on example of applying the net benefit regression framework for cost-effectiveness analysis, which works for both observational and randomized studies with possibly censored data. Many existing methods are special cases within this framework. We provide step-by-step guidance, user-friendly R programs, and examples to illustrate 1) fitting net benefit regressions for possibly censored cost-effectiveness data; 2) implementing doubly robust methods combining net benefit regressions and propensity scores, which may increase the chances to obtain consistent estimates in observational studies; 3) constructing cost-effectiveness acceptability curves; and 4) interpreting the results. The methods in this tutorial are easy to use and lead to more reliable and robust results using typical administrative data, thus providing an attractive option for real-world cost-effectiveness analysis using possibly censored observational data sets.

**Highlights:**

- We illustrate the steps involved in carrying out cost-effectiveness analysis using net benefit regressions with possibly censored demo data by providing step-by-step guidance and code applied to a data set.
- We demonstrate the importance of these new methods by illustrating how naïve methods for handling censoring can lead to biased cost-effectiveness results.

## Background

Clinical studies often encounter challenges associated with right censoring. For instance, at the date of data capture, some patients may have dropped out, been lost to follow-up, or not experienced the event of interest. In such cases, the last date of follow-up is referred to as the censoring time. Patients who are still alive at the time of data capture have costs and survival information available only until their last follow-up times, but their true total costs and full survival time until death are unknown. A recent study of statistical methods used in trial-based economic evaluations found that 15% of methodological studies reviewed described approaches for person-level cost-effectiveness analysis (CEA) with censored data.^1^ The study concluded with a recommendation against naïve methods such as, “(1) Simply ignoring the fact that data are censored, and hence assuming that all patients in the study experience the event of interest, and (2) Treating the data as being complete, and hence simply omitting censored cases from the analyses.” All but 1 methodological study in the review was published more than a decade ago.^1^ This is surprising given the increasing popularity of person-level CEA using “real-world” data; there is a clear need to understand and use methods for censored data in both randomized and observational studies.

While economic decision analytic models (e.g., Markov models) are commonly used to inform funding decision negotiations in many countries and agencies, the analysis of person-level data (e.g., from a data set with cost and effectiveness information from each patient) is also an important research activity, with many such studies published each year. Cost-effectiveness data sets can be assembled from randomized controlled trials, pragmatic studies, or administrative data sources (e.g., surveillance, epidemiology, and end results linked to Medicare administrative and claims data [SEER-Medicare]). These types of data sets, with person-level information on cost and health outcome, are valuable because they allow researchers to estimate the additional gain from new treatments in actual patients and place the estimate in context with the observed incremental costs. However, these sources often contain observational, censored data requiring special care to adjust for the nonrandomized, incomplete nature of the correlated cost and effect data. Without accounting for the unique characteristics of the data, the CEA can provide a flawed assessment of the incremental costs associated with the incremental health outcomes, yielding biased estimates and inaccurate uncertainty assessments. However, there exist methods from statistical CEA that can address the challenges inherent in real-world data. Next, we summarize best practice and provide a hands-on example.

## Main Summary Statistics of Interest in Statistical Cost-effectiveness

The incremental cost-effectiveness ratio (ICER) is a ratio of the incremental cost to the incremental effect, and as a ratio, it has statistical challenges. The incremental net benefit (INB) is not hampered by these shortcomings and provides an important alternative. Both are functions of the cost ( $c_{i}$ ) and clinical effect outcome ( $e_{i}$ ) for the new treatment ( $\mathrm{Tr}t_{i}=1$ ) and usual care ( $\mathrm{Tr}t_{i}=0$ ) ( i=1,…,n for the i th patient). The ICER =ΔC/ΔE and the INB= λΔE−ΔC , where ΔC and ΔE are the differences in the mean cost and mean effectiveness, respectively, and λ is a cost-effectiveness threshold value with various interpretations,^2,3^ such as the decision maker’s unknown willingness-to-pay value for an additional unit of effectiveness. The INB is also the difference between the expected net benefits of new treatment and usual care, where an individual’s net benefit is defined as $nb_{i}=\lambda e_{i}-c_{i}$ .

There are various options for characterizing uncertainty (i.e., variability) in statistical CEA,^4^ including Fieller’s theorem, bootstrap, and the cost-effectiveness acceptability curve (CEAC). The CEAC shows the probability that the new treatment or intervention is cost-effective as a function of the unknown cost-effectiveness threshold value given the data.^5,6^

## Net Benefit Regression with Complete (Uncensored) Data

A well-established method to analyze a cost-effectiveness data set in statistical CEA involves net benefit regression,^7–9^ which refers to a regression equation with net benefit ( $nb_{i}=\lambda e_{i}-c_{i}$ ) as the dependent variable and the treatment indicator variable ( $\mathrm{Tr}t_{i}$ ) and possibly other covariates (and potentially their interactions with treatment) as independent variables. The ability to study cost-effectiveness controlling for other covariates is a major strength of placing CEA in a regression framework. The use of covariate adjustment is essential for observational studies, since treatment assignment or receipt is likely related to covariates, leading to potential confounding issues. In clinical trials, covariates can be used to adjust for imperfect randomization and to improve efficiency.^10^ In addition, detecting policy-relevant interactions (e.g., treatment by covariate) can help identify important subgroups with heterogenous cost-effectiveness through hypothesis-generating analyses.

The above concepts can be formalized with equations by including the treatment indicator $\mathrm{Tr}t_{i}$ , $Z_{i}=(Z_{i1},\ldots ,Z_{\mathrm{ip}})\prime$ as a vector including p covariates (e.g., patient demographics), and possibly $\mathrm{Tr}t_{i}\times Z_{i}$ as the treatment-covariate interactions. For a given cost-effectiveness threshold value of λ, the model is

$$nb_{i}=\beta_{0}+\beta_{\mathrm{Trt}}\mathrm{Tr}t_{i}+\beta \prime_{Z}Z_{i}+\beta \prime_{\mathrm{TrtZ}}\mathrm{Tr}t_{i}\times Z_{i}+\varepsilon_{i}$$

where $\varepsilon_{i}$ is the error term without a specified distribution. Large and statistically significant elements in $\beta_{\mathrm{TrtZ}}$ for an interaction term point toward important patient subgroups, which is helpful for tailoring decision making. Without censoring, the coefficients can be estimated by ordinary least squares (OLS) (for a given λ), and the standard error can be estimated by bootstrapping or the Huber-White robust estimator.^11^ The CEAC can be constructed from net benefit regression,^7^ reflecting the probability that $\beta_{\mathrm{Trt}}>0$ for being cost-effective (in a Bayesian sense). The influence of skewness on statistical inference for the net benefit regression method was evaluated in simulation studies,^12^ which concluded, “Apart from the confidence intervals for treatment effect being a little conservative (i.e., a little too wide), there appears to be no real cause for concern, even when cost data are log-normal and the total sample size is as small as 100.”

## Cost-effectiveness with Censored Data

As noted earlier, studies that capture or record the data before complete cost and effectiveness data are available face challenges associated with right censoring, with their true total costs and full survival time until death possibly unknown. It is tempting to simplify the analysis by using simple methods to handle censoring, such as using complete-case data only (i.e., discarding patients without complete data) or using all data ignoring censoring status (i.e., using the observed data at the last follow-up). Although there exist rare special situations in which the ICER and INB could be unbiased with such naïve methods, in general these simple methods are bias prone.

**Figure 1:**
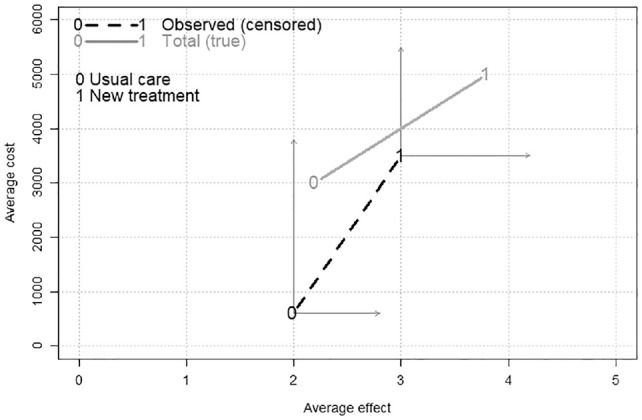
Sample averages for cost and effect for treatment options Trt = 0 (usual care as control) and Trt = 1 (new treatment), indicated with a “0” or “1,” respectively. By using observed data ignoring censoring, the naïve observed incremental cost-effectiveness ratio (ICER) is the slope of the dashed line. In contrast, the true data (with nothing left out) produce an ICER shown as the slope of the solid line. The vertical and horizontal arrows emanating from the observed 0 and 1 connected with the dashed line show that the true average total cost and effect data may be greater or equal (due to further including cost and effect after censoring), indicated by the 0 and 1 points connected by the solid line, whose slope shows the true ICER. For a purpose of illustration, this figure used hypothetical data, assuming that the control and new treatment groups have true average effects of 2.2 and 3.8 and true average costs of 3,000 and 5,000, respectively. Due to censoring, high costs before death are not fully observed. We assume the control and new treatment groups have the average observed effects of 2 and 3 and average observed costs of 600 and 3,500, respectively.

The relevant impact of censoring is related to how it affects estimates of ΔC and ΔE . Figure 1 shows the sample averages for observed cost and effect (e.g., survival time) for treatment options Trt=0 and Trt=1 (indicated with “0” or “1,” respectively). By using observed data ignoring censoring, the naïve observed ICER is the slope of the dashed line. Censoring means the “true” estimates of expected cost and effect will likely be to the northeast of the observed estimates because the full cost (and effect) will almost always be greater (or equal) to the observed cost (and effect) at the censoring time. The impact of censoring on the ICER is illustrated by the difference in slopes between the dashed and the solid connecting lines (with the true ICER equaling the slope of the solid line in Figure 1).

As the different ICERs in Figure 1 suggest, ignoring censoring is often not a good option. For example, if the rate of cost accumulation increases around death, and costs at this time are censored, then costs that are unobserved are both different and important. Because health care costs tend to rise dramatically prior to death, it is incorrect to assume the rate of cost accumulation is similar for the period that is observed (before censoring) and the period that is not observed (censored).^13^ Supplementary Figure S1A demonstrates low coverage probabilities for 95% confidence intervals due to bias from using naïve methods (e.g., complete-case data only [CC] and using all data but ignoring censoring status [AL]), based on simulations performed by Chen and Hoch.^8^

## Potentially Informative Censoring

Statistical methods to address censoring depend on whether the data are subject to informative or noninformative censoring. Most common survival analysis methods (e.g., the Kaplan-Meier method and Cox proportional hazards model) assume censoring is noninformative (i.e., the survival time is independent of censoring time). However, costs and effectiveness such as quality-adjusted life-years (QALYs) are likely to be subject to informative censoring.^14^ For example, a healthier patient will accumulate costs more slowly (with higher quality of life), with less costs (and higher QALYs) both at the censoring time and at the potential death time, leading to informative censoring for cost and QALYs.^15^ Consequently, standard survival analysis techniques are not valid to analyze costs and QALYs directly on the cost (or QALY) scale.^14,16^ According to Lagakos,^17^“when the amount of censoring is small, very little bias is likely to result from the use of methods based on noninformative censoring. However, when informative censoring is extensive, substantial biases can occur, and so informative censoring models should be considered.”

## Cost-effectiveness Analysis with Censored Observational Data

While using administrative data sets can boost sample size and produce real-world evidence, they are not randomized. For example, Ali et al.^18^ assessed the cost-effectiveness of breast-conserving surgery plus hormonal therapy with or without radiotherapy, using SEER-Medicare data, helping to inform the allocation of cancer care resources optimally by generating real-world evidence about the incremental costs of new treatments in relation to their better effectiveness. Their results, based on a real-world data set, suggested that the combination of radiotherapy and hormonal therapy could be cost-effective from a US Centers for Medicare and Medicaid Services perspective.

Although propensity scores and standard survival analysis techniques are often used to evaluate survival using censored observational data, censoring in costs (and QALYs) are more likely to be informative and hence standard methods for noninformative censoring are not recommended. Performing a proper CEA using statistical methods for valid estimation and inference is not very commonplace for such nonrandomized censored data. Various methods for censored CEA^14,19–33^ have been proposed, providing a good foundation, yet there is room for improvement (e.g., estimates are not adjusted for covariates, not efficient, not doubly robust, and not straightforward to construct a CEAC). This article illustrates net benefit regression for censored cost-effectiveness data from observational studies,^8^ which overcomes the aforementioned disadvantages as well as unifying many methods as special cases (e.g., the methods work for uncensored data and unadjusted analysis).^8^ In the following sections, we introduce the methods and provide step-by-step instructions with R programs to illustrate 1) running net benefit regressions for censored cost-effectiveness data, 2) performing doubly robust methods that combine net benefit regressions and propensity scores, 3) constructing CEACs, and 4) interpreting the results.

## Methods for Censored Net Benefit Regression: The Simple Weighted Estimator

Censored net benefit regression^8^ produces consistent estimates when handling censoring using inverse probability of censoring weighting.^34,35^ The simple weighted (SW) estimator uses total costs (and effectiveness) from patients with complete follow-up only, with the weights to represent potential patients that might have been observed. However, the SW estimator does not use cost/effectiveness “history” and wastes information from censored patients (e.g., the observed costs until censoring are ignored in the analysis).

## Methods for Censored Net Benefit Regression: The Partitioned Estimator

When cost/effectiveness history (e.g., the monthly or yearly costs and QALYs) is available, the SW estimator can be improved through partitioning into smaller time “buckets.” For example, if the data are grouped into yearly data “buckets,” for patients censored after the k th year, their costs and effectiveness within the first k years are complete. Therefore, we can fit censored net benefit regression for each year separately, using the yearly cost and effectiveness data. The final partitioned (PT) regression coefficients estimates are the summation of coefficients estimates across all years from the yearly net benefit regressions.^8^

As such, net benefit regression can be used when carrying out CEA with censored data. If only the total costs and effectiveness are available, the SW estimator is useful. If the cost and effectiveness history for different periods is also available, the PT estimator is generally more efficient than the SW estimator is. Both the SW and PT methods are consistent in the statistical sense, with coverage probabilities that are close to the nominal level of 95% in simulation (Supplementary Figure S1B).^8^

## Methods for Observational Data: Propensity Scores and Doubly Robust Methods

Define the causal average INB as $\delta =E\left(nb^{\left(1\right)}\right)-E(nb^{\left(0\right)})$ , where $nb^{\left(1\right)}$ and $nb^{\left(0\right)}$ are an individual’s potential outcomes of net benefits (one actual and one counterfactual) if the corresponding treatment Trt were 1 and 0 (e.g., new treatment v. usual care), respectively. Since an individual can only receive 1 treatment option, only $nb^{\left(1\right)}$ or $nb^{\left(0\right)}$ is observed for each person. In administrative data, treatment assignment is not random, leading potentially to selection bias, where patient characteristics are associated with the likelihood of receiving treatment and with the outcomes.

Both propensity scores and doubly robust methods are modern causal methods, which are useful for estimating causal average treatment effects ( δ in our case).^36^ Propensity score methods^37–39^ provide ways to balance measured covariates across treatment and comparison groups. The augmented inverse propensity weighted estimator^40,41^ combines a regression-based estimator and a propensity score–weighting method (inverse probability of treatment weighting) and is a “doubly robust” method in that it requires either the propensity or the outcome model to be correctly specified, but not necessary both.

## Censored Net Benefit Regression with Observational Data: A Doubly Robust Estimator

When the net benefit regression (without interaction) is correctly specified, the causal average INB ( δ ) equals $\beta_{\mathrm{Trt}}$ in the net benefit regression. However, estimates are prone to bias if the net benefit regression model is misspecified. Likewise, if the propensity score model is not modeled correctly, bias may also be introduced. To diminish the possible bias in estimating “causal average INB” due to misspecified models, the doubly robust method^8^ combines both censored net benefit regressions and propensity scores and remains consistent when either the propensity score model or the net benefit regression model is correctly specified. This property may increase the chances to obtain consistent estimates in observational studies, and it can always obtain consistent estimates in randomized trials (since the propensity score is known). Table 1 summarizes the benefits of using the doubly robust method in randomized and observational studies,^8,24^ supporting the contention that the doubly robust method is a useful tool for CEA, especially for administrative data with nonrandomized treatment assignment and possibly censored outcomes.

**Table 1:** Advantages Regarding Statistical Consistency (Asymptotically Unbiasedness) of the Doubly Robust Method Combining Net Benefit Regression and Propensity Scores in Randomized and Observational Studies

Study Type	Censored Data	NBR Correctly Specified	Propensity Correctly Specified	Asymptotically Unbiased Estimate Benefit from
Randomized	No	Yes	N/A	No advantage
		No	N/A	Robust for misspecification (NBR)
	Yes	Yes	N/A	Adjusting for censoring
		No	N/A	Adjusting for censoring and robust for misspecification (NBR)
Observational	No	Yes	Yes	No advantage
			No	Robust for misspecification (propensity)
		No	Yes	Robust for misspecification (NBR)
			No	No advantage
	Yes	Yes	Yes	Adjusting for censoring
			No	Adjusting for censoring and robust for misspecification (propensity)
		No	Yes	Adjusting for censoring and robust for misspecification (NBR)
			No	No advantage

NBR, net benefit regression; N/A, not applicable (since treatment is randomly assigned and propensity score is completely known in randomized studies, the consistency is guaranteed; note that propensity scores and doubly robust methods could still be used to improve efficiency in randomized studies).

## Data

To illustrate the methods, we simulated a cost-effectiveness data set mimicking administrative data including 2,000 hypothetical patients with cardiovascular disease, motivated by a real cardiovascular study. The data-generation process is similar to the simulation scenario in Chen and Hoch^8^ with these main differences: 1) more covariates are involved (increase from 1 to 3 covariates) and 2) in addition to using life-years as effectiveness, QALYs are also generated based on a heart failure event (i.e., a patient’s quality of life tends to be lower after heart failure). More details of the data generation are provided in the Supplementary Materials. For this simulated data set, we know the true (uncensored and counterfactual potential) means based on statistical theory (Supplementary Table S1) and thus the true INBs, which allows us to evaluate the performance of the different methods.

The first few rows of the data are shown in Table 2. The *survival* variable is the follow-up time (in years) since the enrollment into the study. The *dead* variable is mortality indicator with “1” for the occurrence of death (i.e., complete data) and “0” indicating censoring (i.e., time until death is unknown but was known to be alive at the time indicated by *survival*). The *Trt* variable indicates the new treatment group (*Trt* = 1) versus the usual care comparison group (*Trt* = 0). Patients who are younger, with left bundle branch block (LBBB) conduction disturbance are more likely to receive the new treatment than the comparison. The *Age65*, *LBBB*, and *Female* variables are binary baseline covariates, with “1” for age ≥ 65 y, LBBB, and female, respectively, and “0” otherwise. U-shaped costs (high initial and terminal costs) were generated and grouped into yearly observed cost summary variables called *cost.1*, *cost.2*, and all the way up to *cost.15* (in $1,000s). In our example, the new treatment has higher initial costs for performing the treatment but lower annual costs subsequently. We used life-years (LYs) and QALYs as outcome measures of effectiveness and grouped QALYs into 15 annual summary variables as well. The patient health-related quality-of-life (QOL) was simulated in each of the yearly intervals, ranging from 1 for good health to 0 at death, and each yearly QALY was calculated as the integration of QOL within that year (see Supplementary Figure S3 for more illustration). When the variables *cost.1* to *cost.15* (and *QALY.1* to *QALY.15*) are summed, they provide the total observed costs (and QALYs) over 15 y, named *tot.cost* (and *tot.QALY*), respectively.

**Table 2:** First 3 Rows of the Simulated Data^a^

ID	Survival	Dead	Trt	Age65 y	LBBB	Female	cost.1	…	cost.15	tot.cost	QALY.1	…	QALY.15	tot.QALY
1	4.10	0	0	0	0	1	6.272	…	0	9.585	0.63	…	0	2.43
2	3.52	0	1	0	1	1	15.375	…	0	15.773	0.52	…	0	2.21
3	3.78	1	1	0	0	0	13.669	…	0	37.328	0.55	…	0	2.40

a Costs are in $1,000s, and survival and quality-adjusted life years (QALYs) are in years.

In this data set, costs and QALY data were collected up to 15 y; however, the longest follow-up time was slightly less than 15 y. Therefore, a smaller time limit should be chosen (e.g., 10 y), and time-restricted mean survival time/QALY/costs^42,43^ are used (more details in the following section). A consequence of applying such a restriction is that a patient alive at 10 y is considered as having complete data. This leads to a censoring rate of 49% within the 10-y horizon in this data set.

## Steps Involved in Performing Net Benefit Regression Methods for Censored Observational Data

The theory behind censored net benefit regression and the doubly robust method have been described elsewhere.^8,9^ Here, we focus on the steps involved in performing net benefit regression methods for an administrative cost-effectiveness data set. R programs are provided for the main steps, and more examples and technical details are available in the Supplementary Materials.

### Step 1: Preparation

#### Load required package

The first line loads the NetBenReg package to perform net benefit regression. Before using this package, the NetBenReg package needs to be installed in R (see Supplementary Materials for details to install this package in R).

library(NetBenReg)

#### Load data

The next line loads the data set CEdata.

data(CEdata)

#### Choose time limit

Due to censoring, the right tail of the distribution of survival time cannot be estimated reliably, leading to unstable estimation of the mean survival/QALY/costs.^44^ For instance, if all patients are followed for up to 10 y, the cost after 10 y is not observed, and thus, estimating mean costs after that time is not reliable. Consequently, researchers often turn to the time-restricted mean survival time/QALY/costs.^42,43^ For example, we can measure survival/QALY/costs saved within a time horizon of *L* years, where *L* is chosen such that a “reasonable” number of subjects are still being observed at that time (e.g., choose *L* as the upper quartile of the follow-up times). This means that we are interested in outcomes accumulated until death or up to *L* years, whichever occurs first. Although our data set provides the costs observed up to 15 y, the longest follow-up time is 14.99 y, meaning that no patients were observed at the end of 15 y. Therefore, a smaller time limit *L* (e.g., 10 y with sufficient data using an intuitive cutoff) could be chosen, and patients alive at 10 y have complete data. This time limit is required for both SW and PT methods. Patients censored within *L* years are still considered as censored within this time horizon, which can be handled by the SW and PT methods appropriately. In the Supplementary Materials, we provide examples showing error or warning messages in R when we choose too large an *L*.

#### Choose cost-effectiveness threshold values

To perform net benefit regression, we need to choose a few cost-effectiveness threshold values denoted by λ. A sequence of λ values from 0 to multiple times of the ICER is a good start. We can first fit covariate-adjusted regressions using the SW method to estimate a covariate-adjusted ICER ($2,680/QALY, see step 2). Based on this, we can create a cost-effectiveness threshold sequence of $ 0, $500, $1,000, . . ., $6,000 for 1 additional QALY:

lambda=seq(0,6,0.5)

This line generates lambda as a sequence from 0 to 6 (in $1,000s), with 0.5 as the increment of the sequence (this creates a range from $ 0 to $6,000 jumping by $500 increments).

### Step 2: Fitting Censored Net Benefit Regressions

We can fit a covariate-adjusted net benefit regression using the SW method with QALY as effectiveness:

fit1<-NetBenReg(Followup=CEdata$survival, #follow-up time 

 delta=CEdata$dead,  #1=complete,0=censor

 group=CEdata$Trt,   #1=treatment, 0=comparison

 Cost=CEdata[,8:22],  #a matrix of cost history

 Eff=CEdata[,24:38],  #a matrix of effectiveness history

 Part.times=1:15,   #end timepoints of intervals 

 Method=‘SW’,   #use Simple Weighted method

 Z=CEdata[,5:7],   #adjust covariates in regressions

 Eff.only=TRUE,   #also fit effect-only regression

 Cost.only=TRUE,   #also fit cost-only regression

 lambda=lambda,   #cost-effectiveness threshold

 L=10)     #time limit

Print(fit1)

The first line clarifies that the variable *survival* in the data set CEdata (described in “Data” section) denotes the follow-up time for each patient. The next line delta=CEdata$dead indicates if an individual was complete (1) or censored (0) after the survival time stopped being recorded. The line group=CEdata$Trt clarifies if the individual received new treatment (1) or usual care (0). Below that are the lines Cost=CEdata[,8:22] and Eff=CEdata[,24:38] clarifying the 15 columns for yearly cost and effect data. The line Part.times=1:15 specifies the end time point as 1, 2, . . ., 15, meaning that the intervals are [0, 1], (1, 2], . . ., (14, 15] years for each grouped cost and effectiveness variable. The option Method=‘SW’ requests the SW method. We can also fit the same model using the PT method with Method=‘PT’. Covariates, introduced through Z=CEdata[,5:7], consist of a data frame of the variables*Age65*, *LBBB*, and *Female* stored in columns 5, 6, and 7 of CEdata. As a special case, the option Z can be removed if no covariates are desired for adjustment (e.g., in randomized studies). The options Eff.only=TRUE and Cost.only=TRUE additionally fit the regressions with effect and cost as dependent variables, respectively. The final 2 lines lambda=lambda and L=10 refer to the cost-effectiveness threshold and the time horizon of *L* years described above.

To explore heterogeneous cost-effectiveness and identify subgroups, we can further include treatment-covariate interactions. The option interaction= specifies the names of covariates having interaction with treatment. For example, the following code adds a treatment-LBBB interaction (bold in following code):

fit2<-NetBenReg(Followup=CEdata$survival, delta=CEdata$dead, group=CEdata$Trt, Cost=CEdata[,8:22], Eff=CEdata[,24: 38], Part.times=1:15, Method=‘PT’, Z= CEdata[,5:7], **interaction=c("LBBB")**, Eff.only=TRUE, lambda=lambda, L=10)

#### Caution

Two naïve methods are also allowed (Method=‘CC’ for complete-case only; Method=‘AL’ for all data ignoring censoring). However, they are not recommended for censored data due to bias. Without censoring, the 4 methods (SW, PT, CC, AL) are equivalent to OLS.

#### Interpretation

Table 3 summarizes the results. To evaluate different methods, we used several methods to obtain “true” ICERs and INBs: 1) the “Theory” method is based on the true mean values analytically derived by statistical theory, leading to the overall ICER of $2,750/QALY (=$2,713/0.99 QALY) in the whole population; 2) the “OLS using uncensored data” method is based on the simulated uncensored outcomes (including cost and effect after censoring time); 3) the “Using true outcomes” method is based on the simulated uncensored potential outcomes (counterfactuals), leading to an overall ICER of $2,501/QALY (=$2,585/1.03 QALY). Although the estimates from the methods (2) and (3) will approach the mean values from the method (1) when the sample size approaches infinity, the 3 methods could be slightly different with smaller sample sizes, as in our example. Methods (2) and (3) might be better “true” values, since our data set is a censored version of the full uncensored data set that was used to calculate (2) and (3).

**Table 3:** Coefficient Estimates (Standard Errors) (in $1,000s) and *P* Values for Treatment and Interaction Terms in Net Benefit Regressions with Different Values of Cost-Effectiveness Threshold λ (in $1,000/QALY) for the Hypothetical Data Using Quality-Adjusted Life-Years (QALYs) as Effectiveness, Limited to a 10-y Time Horizon^a^

Method	Model Term	Interpretation	λ=0	λ=3	λ=6	λ= NA (Effect Only)
			Est (SE) [*P* Value]	Est (SE) [*P* Value]	Est (SE) [*P* Value]	Est (SE) [*P* Value]
**Covariate-adjusted net benefit regression (without interaction)**						
(Naïve) CC	Treatment v. control	Covariate-adjusted INB	−3.34 (0.43) [<0.001]	−0.75 (0.60) [0.21]	1.85 (0.93) [0.048]	0.86 (0.14) [<0.001]
(Naïve) AL			−3.80 (0.30) [<0.001]	−2.03 (0.38) [<0.001]	−0.26 (0.59) [0.65]	0.59 (0.09) [<0.001]
SW			−2.55 (0.39) [<0.001]	0.30 (0.62) [0.63]	3.14 (1.01) [0.002]	0.95 (0.15) [<0.001]
PT			−2.49 (0.36) [<0.001]	0.46 (0.53) [0.39]	3.41 (0.82) [<0.001]	0.98 (0.12) [<0.001]
OLS using uncensored data			−2.42 (0.29) [<0.001]	0.67 (0.44) [0.126]	3.77 (0.70) [<0.001]	1.03 (0.10) [<0.001]
**Covariate-adjusted net benefit regression with treatment-LBBB interaction**						
PT	Treatment v. Control	Non-LBBB subgroup’s covariate-adjusted INB	−3.24 (0.50) [<0.001]	−2.43 (0.71) [<0.001]	−1.61 (1.11) [0.145]	0.27 (0.16) [0.094]
	Treatment-LBBB interaction	Difference between LBBB’s and non-LBBB’s covariate-adjusted INBs	1.52 (0.72) [0.036]	5.94 (1.04) [<0.001]	10.36 (1.61) [<0.001]	1.47 (0.23) [<0.001]
OLS using uncensored data	Treatment v. Control	Non-LBBB subgroup’s covariate-adjusted INB	−3.17 (0.41) [<0.001]	−2.69 (0.58) [<0.001]	−2.21 (0.91) [0.015]	0.16 (0.14) [0.24]
	Treatment-LBBB interaction	Difference between LBBB’s and non-LBBB’s covariate-adjusted INBs	1.53 (0.58) [0.008]	6.88 (0.85) [<0.001]	12.22 (1.34) [<0.001]	1.78 (0.19) [<0.001]
**Doubly robust method (covariate-adjusted regression with interaction + propensity scores)**						
DR-PT	Treatment v. Control	Causal average INB	−2.43 (0.37) [<0.001]	0.66 (0.53) [0.21]	3.75 (0.83) [<0.001]	1.03 (0.12) [<0.001]
Using true outcomes			−2.58	0.52	3.62	1.03
Theory			−2.71	0.25	3.21	0.99

INB, incremental net benefit; LBBB, left bundle branch block; QALY, quality-adjusted life-years.

a Est is the coefficient estimate (in $1,000s) for treatment (dummy variable) and treatment-LBBB interaction from net benefit regressions or causal average INB from doubly robust methods; coefficients for other covariates are omitted. SE is the estimated standard errors. Covariate-adjusted INB is estimated by the coefficient for treatment (dummy variable) in covariate-adjusted regressions without interactions. Non-LBBB subgroup’s covariate-adjusted INB is estimated by the coefficient for treatment in covariate-adjusted regressions with the treatment-LBBB interaction, and difference between LBBB’s and non-LBBB’s covariate-adjusted INBs is estimated by the coefficient for treatment-LBBB interaction in the same regressions, and LBBB subgroup’s covariate-adjusted INB can be calculated by their summation. When λ = 0, the dependent variable = −1 × cost, equivalent to the cost-only regression by switching the signs of estimated coefficients. For λ = NA, the dependent variable = effect (does not belong to net benefit regressions). PT is the partitioned net benefit regression using cost and QALY history. SW is the simple weighted net benefit regression. (Naïve) CC is the regression using complete-case (uncensored) cases only. (Naïve) AL is the regression using all data ignoring censoring status. For the uncensored data, the 4 methods (SW, PT, CC, AL) are equivalent to ordinary least squares (OLS). DR-PT is the doubly robust method with the partitioned method. Results from OLS using uncensored data and using true outcomes are based on the generated uncensored (and potential counterfactual outcomes) in the data simulation process, and results from theory are based on the true mean values analytically derived by theory. These 3 methods are used as true values to evaluate performance of other methods.

For covariate-adjusted regression without interaction, the coefficient for the treatment is the adjusted INB. For example, when the cost-effectiveness threshold is $6,000/QALY, the estimated covariate-adjusted INB is $3,410 by the PT method. When λ = 0, the dependent variable is −1 × cost, equivalent to the cost-only regression with switched signs for the estimated coefficients; for the effect-only model, the dependent variable = effect (indicated as λ = NA in Table 3). Then it is easy to obtain a covariate-adjusted ICER: for example, the ICER is $2,680/QALY using the SW method (=$2,550/0.95 QALY as the ratio of the treatment coefficient estimates in cost-only and effect-only regressions). Comparing the estimates from covariate-adjusted regressions using the 4 methods to the true values (“OLS using uncensored data” method) in Table 3, it is apparent that estimates of both naïve methods are far away from the true values, with the CC method less biased than the AL method in this example. Both SW and PT methods produce similar estimates to the true values, but the PT method is generally more efficient, with 8% to 20% reductions in standard errors by using cost and QALY history.

The coefficient for the treatment-LBBB interaction is significantly positive for effect and all cost-effectiveness threshold λ values, indicating that effect as well as cost-effectiveness are heterogeneous across LBBB status. LBBB patients achieve significantly higher INBs from the new treatment as compared with their non-LBBB counterparts, adjusting for other covariates. The coefficients for the treatment (main effect) and treatment-LBBB interaction can be used to calculate adjusted INBs in LBBB and non-LBBB subgroups, respectively. For example, when the cost-effectiveness threshold is $6,000/QALY, the regression adjusted INB is −$1,610 for the non-LBBB subgroup and $8,750 (=$10,360–$1,610) for the LBBB subgroup. This result provides evidence that the cost-effectiveness of the new treatment can vary by LBBB status.

### Step 3: Using a Doubly Robust Method to Estimate Causal Average INB with Observational Data

#### Propensity scores

The propensity score is the probability of receiving the new treatment, which could be estimated by fitting a logistic regression (or other models) with treatment indicator as the dependent variable and the potential confounders as explanatory variables. Generally, if a variable is thought to be related to the outcome but not the treatment, including it in the propensity score (for which it is an irrelevant variable) should reduce bias.^45^ More practical guidelines about choice of variables in propensity score models can be found elsewhere.^45–48^

#### Implementing the doubly robust method

We can perform the doubly robust method by further specifying the option Doubly.Robust=TRUE to request the doubly robust method and the option PS.Z=CEdata[,5:7] to include the 3 covariates into the propensity score model using logistic regression.

fit3<-NetBenReg(Followup=CEdata$survival, delta=CEdata$dead, group=CEdata$Trt, Cost=CEdata[,8:22], Eff=CEdata[,24: 38], Part.times=1:15, Method=‘PT’, Z= CEdata[,5:7], interaction=c("LBBB"), **PS.Z=CEdata[,5:7], Doubly.Robust=TRUE**, Eff.only=TRUE, lambda=lambda, L=10)

#### Caution

We can examine whether there exists extreme propensity scores close to 0 or 1 (program in the Supplementary Materials), which is important because extreme propensity scores may lead to huge weights and hence unstable results. Crump et al.^49^ suggested a rule of thumb of thresholds of 0.1 and 0.9 for extreme propensity scores. By default, the function NetBenReg replaces the propensity scores smaller than 0.1 by 0.1 (and greater than 0.9 by 0.9). It also produces warning messages when there are estimated propensity scores outside [0.1, 0.9]. Supplementary Figure S2 shows that the range of estimated propensity scores are sufficiently overlapping across treatment groups without extreme values, indicating that patients in the 2 groups are comparable after propensity score adjustment.

#### Interpretation

The doubly robust method estimates causal average INB, which is causal average treatment effects on net benefit for the entire population (those who did and did not receive the new treatment). Table 3 summarizes the estimates from the doubly robust method, which are close to the true values.

### Step 4: Constructing CEAC

For net benefit regression without interaction or the doubly robust method, the CEAC can be constructed based on the *P* value ( p ) for ${\hat{\beta }}_{\mathrm{Trt}}$ (or $\hat{\delta }$ for the doubly robust method, where $\delta =E\left(nb^{\left(1\right)}\right)-E(nb^{\left(0\right)})$ is the causal average INB). With a given cost-effectiveness threshold λ value, the vertical axis for the CEAC is p/2 if ${\hat{\beta }}_{\mathrm{Trt}}<0$ or 1−p/2 if ${\hat{\beta }}_{\mathrm{Trt}}>0$ (i.e., 1-sided *P* value of ${\hat{\beta }}_{\mathrm{Trt}}$ ).^7^ For net benefit regressions with interactions, the cost-effectiveness is heterogeneous, and hence, subgroup-specific CEACs can be constructed.^8^ Note that the most common interpretation of a CEAC is in a Bayesian framework. Thus, the 1-sided *P* value can be interpreted as the “probability that the intervention is cost-effective” due to its equivalence to Bayesian analysis incorporating a noninformative prior.^50^ The impact of informative priors on CEACs has been discussed elsewhere.^51^

#### Interpretation

**Figure 2:**
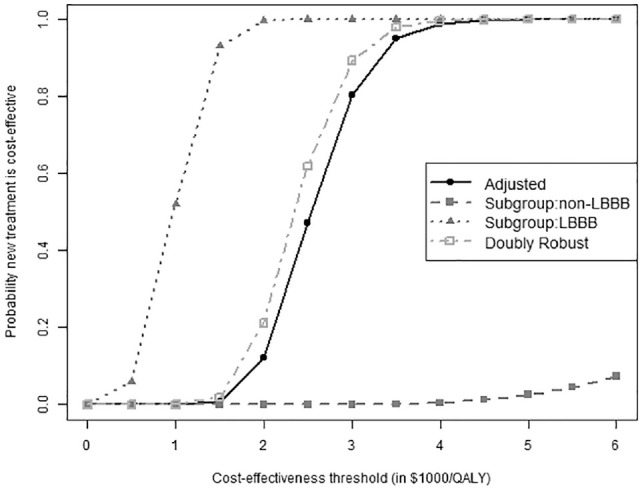
The cost-effectiveness acceptance curves (CEACs) for the hypothetical data limited to a 10-y time horizon, using quality-adjusted life-years (QALYs) as effectiveness with different values of cost-effectiveness threshold. The black solid curve is from covariate-adjusted partitioned regression. The gray dotted and dashed curves are for left bundle branch block (LBBB) and non-LBBB patients, respectively, based on the partitioned regression including treatment by LBBB interaction and adjusting for other covariates (age, gender, in addition to LBBB). The gray dash-dotted curve is from the doubly robust method (combining covariate-adjusted regression with an LBBB-treatment interaction and propensity scores).

Figure 2 demonstrates 4 CEACs based on the fitted models (made using the program in the Supplementary Materials), showing the probabilities that the new treatment is cost-effective compared with the comparison group at different cost-effectiveness threshold values (a sequence of $ 0, $500, . . ., $6,000 chosen in step 1). Among LBBB patients, the probability that the new treatment is cost-effective is much higher than the probability among non-LBBB patients, indicating heterogeneous cost-effectiveness across LBBB status. This was foreshadowed by the significant treatment-LBBB interaction observed earlier.

## Conclusions

Funding negotiations are often informed by research evidence introduced through a health technology assessment (HTA) process. As the field of HTA begins to embrace real-world evidence to address well-known limitations in randomized trials, there will be demand for CEA conducted using person-level administrative data. Even in the United States, increases in available cost and health outcome data combined with Medicare’s new capabilities to consider drug costs as well as their effectiveness suggest that CEA may be a very strategic part of future comparative effectiveness research intent on informing health care funding decisions. In addition, if approvals for new drugs continue to outpace the evidence,^52^ the demand for real-world evidence about value (i.e., cost and effectiveness) promises to continue to grow.

Therefore, applying state-of-the-art methods to analyze censored observational data in a net benefit regression framework is essential. When cost-effectiveness data are censored, naïve methods to handle censoring should be avoided, especially for heavily censored data. The doubly robust method combines net benefit regressions and propensity scores in an easy-to-use manner, leading to more reliable results for observational data with censored costs or health outcomes. In addition, the methods can be applied to randomized clinical trials as well. This provides a strong option for CEA using possibly censored data from observational and randomized studies. With the methods illustrated in this article, potential challenges due to nonrandomized and censored data can be addressed in a sound manner.

## Supplemental Material

sj-docx-1-mdm-10.1177_0272989X241230071 – Supplemental material for A Tutorial on Net Benefit Regression for Real-World Cost-Effectiveness Analysis Using Censored Data from Randomized or Observational Studies Supplemental material, sj-docx-1-mdm-10.1177_0272989X241230071 for A Tutorial on Net Benefit Regression for Real-World Cost-Effectiveness Analysis Using Censored Data from Randomized or Observational Studies by Shuai Chen, Heejung Bang and Jeffrey S. Hoch in Medical Decision Making
