# Supplementary material for: A Tutorial on Net Benefit Regression for Real-World Cost-Effectiveness Analysis Using Censored Data from Randomized or Observational Studies
Source: Med Decis Making. 2024 Feb 12;44(3):239–51. doi: 10.1177/0272989X241230071 (PMC10987289; doi:10.1177/0272989X241230071)
Supplement: sj-docx-1-mdm-10.1177_0272989X241230071 – Supplemental material for A Tutorial on Net Benefit Regression for Real-World Cost-Effectiveness Analysis Using Censored Data from Randomized or Observational Studies [file sj-docx-1-mdm-10.1177_0272989X241230071.docx]

**Supplementary Materials for “A Tutorial on Net Benefit Regression for Real World Cost-Effectiveness Analysis Using Censored Data from Randomized or Observational Studies”**

**Supplementary results**

**Supplementary Figure S1**. Coverage percentage probabilities for 95% confidence intervals of incremental net benefit (INB) using various methods by differing sample size (n), cost-effectiveness threshold value (*λ*, in $1000s), and censoring rates (H is for a heavy censoring rate 48%, L is for a light censoring rate 25%), based on 2,000 simulation runs. Panel (A) is for net benefit regressions with naïve methods for handling censoring using complete-case data only (CC) or using all data ignoring censoring status (AL). Panel (B) is for the censored net benefit regressions without using cost history (SW) and using the partitioned method to incorporate cost history (PT). The vertical dashed line is the nominal coverage (95%).

**(A)**


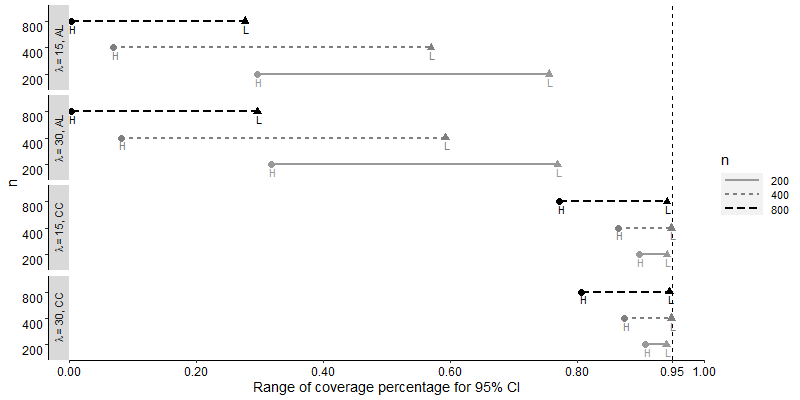


**(B)**


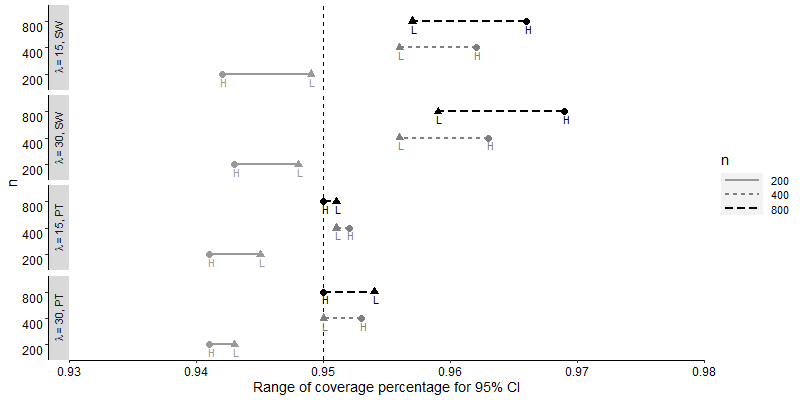


**Supplementary Figure S2**. The histograms of the estimated propensity scores in the doubly robust method for the hypothetical data. The upper panel is for the comparison group ($Trt$ = 0) and the lower panel is for new treatment group ($Trt$ = 1). Due to the fact that only two binary covariates (*age65* and *LBBB*) are significantly associated with treatment assignment, the estimated propensity scores are discrete with 4 levels.


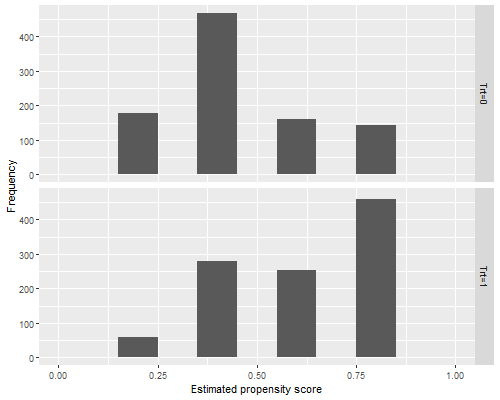


**Supplementary Table S1. True mean outcome values and true incremental cost-effectiveness ratio (within 10 years) of the simulated data for each covariate subgroup, analytically derived by statistical theory.**

|  |  |  | ***LY*** | | ***QALY*** | | ***Cost (in $1,000s)*** | | ***ICER*** | |
| --- | --- | --- | --- | --- | --- | --- | --- | --- | --- | --- |
| ***Age65*** | ***LBBB*** | ***Female*** | ***Trt=0*** | ***Trt=1*** | ***Trt=0*** | ***Trt=1*** | ***Trt=0*** | ***Trt=1*** | ***(in $1,000/LY)*** | ***(in $1,000/QALY)*** |
| 0 | 0 | 0 | 6.82 | 6.94 | 3.06 | 3.24 | 18.258 | 21.612 | 28.061 | 18.399 |
|  |  | 1 | 7.06 | 7.17 | 3.23 | 3.41 | 18.207 | 21.425 | 28.376 | 17.567 |
|  | 1 | 0 | 7.28 | 8.43 | 3.39 | 5.17 | 15.463 | 17.508 | 1.774 | 1.147 |
|  |  | 1 | 7.49 | 8.57 | 3.55 | 5.28 | 15.321 | 17.318 | 1.857 | 1.158 |
| 1 | 0 | 0 | 6.31 | 6.45 | 2.73 | 2.91 | 18.329 | 21.975 | 27.846 | 20.559 |
|  |  | 1 | 6.57 | 6.70 | 2.90 | 3.08 | 18.299 | 21.796 | 27.884 | 19.390 |
|  | 1 | 0 | 6.82 | 8.13 | 3.06 | 4.94 | 15.742 | 17.923 | 1.663 | 1.158 |
|  |  | 1 | 7.06 | 8.29 | 3.23 | 5.06 | 15.604 | 17.710 | 1.710 | 1.147 |

Note: LY, life year; QALY, quality-adjusted life years; ICER, incremental cost-effectiveness ratio. The true average outcomes in overall population can be calculated by weighted average of the means in this table, where the weights are the proportions of patients in each covariate subgroup. The true values in Table 3 of the main text by “Theory” method is then calculated by the difference in the true average outcomes (effects or net benefits).

**Data generation process for the simulated example data**

Covariates

We first generated *X*_1_, *X*_2_, and *X*_3_ from a mean-zero multivariate normal distribution with variance = 1 and covariance = 0.3. We then generate the covariates *Age65*, *LBBB* and *Female* by dichotomizing *X*_1_, *X*_2_, and *X*_3_: *Age65* = I(round(10*X*_1_+60)≥65), *LBBB* = I(*X*_2_>0), and *Female* = I(*X*_3_>0).

Treatment

Treatment indicator *Trt* was generated from a logistic regression with logit{Pr(*Trt* = 1)} = -0.5 - 0.5*Age65* + 1.5*LBBB*, indicating that patients who are younger, with LBBB are more likely to receive the new treatment than the control.

Survival, heart failure, censoring, and follow-up times

Survival time (i.e., uncensored life years) was generated from an exponential distribution with the rate parameter of 1/exp(2.5 − 0.2*Age65* + 0.2*LBBB* + 0.1*Female* + 0.05*Trt* + 0.6*Trt* × *LBBB*), where the positive coefficient for the interaction indicates that the treatment has a better effect to prolong the life years for the LBBB group compared to the non-LBBB group. Survival time was then truncated at 15 years. The patients may also experience heart failure, which will impact their quality-of-life substantially. The heart failure time was generated independently from an exponential distribution with the rate parameter of 1/exp{0.8×(2.5 − 0.2*Age65* + 0.2*LBBB* + 0.1*Female)* + 3×(0.05*Trt* + 0.6*Trt* × *LBBB*)}. Compared to survival time, the heart failure time has bigger coefficients for treatment and interaction, indicating that the treatment has more effect on heart failure prevention than prolonging the life years, especially among LBBB group. The censoring time was generated independently from a uniform distribution on [1, 16] years. The follow-up time is the minimum of survival and censoring times. If the censoring occurs before the death, the true survival time is not observed, leading to a death indicator of 0.

Costs

U-shaped sample paths for the cost distribution were adopted where the entire time period of *L* = 15 years was partitioned into 15 equal intervals. Each individual’s costs consisted of random initial diagnostic costs incurred at time 0, random terminal costs incurred at the death time, fixed annual costs (which vary from individual to individual), and random annual costs (which vary from year to year). The random annual costs and terminal costs for all patients were generated from log-normal distributions with parameters (4, 0.2^2^) and (9, 0.6^2^), respectively. The diagnostic costs were generated from log-normal distributions with parameters (9.5, 0.2^2^) and (8.5, 0.2^2^) for the treatment group (*Trt* = 1) and the control group (*Trt* = 0), respectively. The fixed annual costs were generated from log-normal distributions with parameters (7, 0.2^2^), (6.6, 0.2^2^), (6, 0.2^2^), and (4.5, 0.2^2^) for *LBBB* = 0, *Trt* = 0 group, *LBBB* = 1, *Trt* = 0 group, *LBBB* = 0, *Trt* = 1 group, and *LBBB* = 1, *Trt* = 1 group, respectively. Thus, the new treatment has higher initial diagnostic costs for conducting the treatment, but lower fixed annual costs subsequently. The diagnostic costs were added to the fixed and annual costs in the first year. If the patient were lost during follow-up within a time interval, the observed costs in this year were prorated assuming that diagnostic, fixed, and annual costs were evenly spread in this year.

QALY

For each patient, the quality-of-life (QOL) was generated based on the health status. It consisted of fixed-part (which vary from individual to individual but fixed within each individual) and random-part (which vary from year to year). The fixed-part was generated from a uniform distribution on [0, 0.15]. Two types of the random-part were generated: 1) from a uniform distribution on [0, 0.15], and 2) from a uniform distribution on [0.3, 0.7]. For patients experiencing heart failure, their QOL is the summation of the fixed-part and the type-1 random-part, leading to a QOL within the range of [0, 0.3]. For patients not experiencing heart failure, their QOL is the summation of the fixed-part, and both the type-1 and type-2 random-parts, leading to a QOL within the range of [0.3, 1]. The QOL becomes 0 at death. The yearly QALY was then calculated as the integration of QOL over the survival time within each year. If the censoring occurs before death, the true QALY is not observed, and the observed QALY is calculated up to the follow-up time. Supplementary Figure S3 illustrates the QALY calculated based on QOL for one individual.

**Supplementary Figure S3**. Demonstration of quality-adjusted life years (QALYs) for an individual. The quality-of-life (QOL) drops after the occurrence of heart failure and becomes 0 at death. The total QALY is the total area under the curve (dark gray area plus light gray area). The yearly QALY is the area under the curve within each year. Due to censoring, the observed QALY is the dark gray area only.


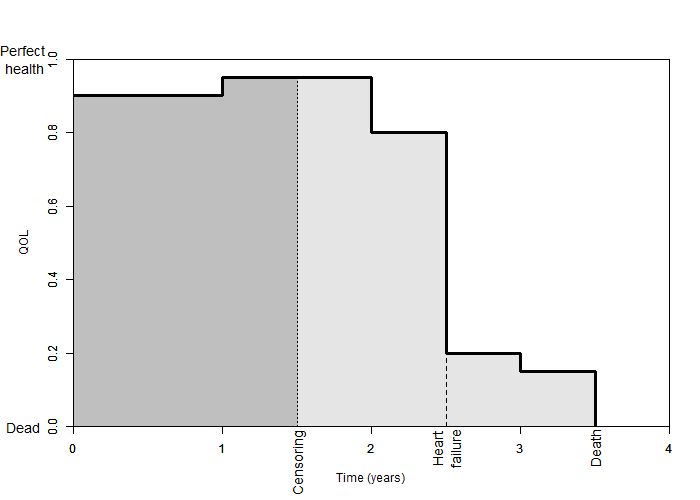


Potential uncensored outcomes (counterfactuals)

During the simulation, we also generate potential uncensored outcomes (counterfactuals). These “true” values can be used by “oracle” methods (i.e., “OLS using uncensored data” and “Using true outcomes” in Table 3 of the main text) to compare different methods. For each patient, the potential uncensored outcome is generated following the process above, except that we generated two sets of outcomes for each patient: Outcome *Y*^(1)^ under treatment (*Trt* = 1), and outcome *Y*^(0)^ under control (*Trt* = 0), where *Y* can be (uncensored) costs, life years, or QALY. The actual (uncensored) outcome is then either *Y*^(1)^ or *Y*^(0)^, depending on the treatment assignment. The “OLS using uncensored data” method used the uncensored *Y* in net benefit regressions with OLS method. The “Using true outcomes” used the sample average of the difference between (uncensored) potential outcomes *Y*^(1)^ - *Y*^(0)^ to calculate the causal average INB.

**Technical details and explanation of R code and output for NetBenReg**

Here we provide the technical details and explanation for the example R programs (tested in R v4.3.1) using function NetBenReg. The R package can also be downloaded from [https://github.com/shuaichencode/NetBenReg](https://github.com/shuaichencode/nbreg).

**Description of** NetBenReg

Estimation of net benefit regression for possibly censored cost-effectiveness data from randomized or observational studies.

**Usage**

NetBenReg(Followup, delta, group, Cost=NULL, Eff=NULL,

Part.times=NULL, Z=NULL, PS.Z=NULL, interaction=NULL,

Method=c('SW','PT','CC','AL'), Sep.K=TRUE, PS.trim=0.1,

Doubly.Robust=FALSE, Eff.only=FALSE, Cost.only=FALSE,

lambda=NULL, L)

**Arguments**

| Followup | vector containing continuous positive follow-up time. |
| --- | --- |
| Delta | vector containing binary indicator of event, 1 - complete, 0 – censored. |
| group | vector containing binary treatment indicator, 1 - treatment, 0 – control. |
| Cost | vector, matrix, or dataframe containing observed total or grouped costs (cost history), Cost[i,j] is observed cost of the ith people accumulated in the jth interval. |
| Eff | vector, matrix, or dataframe containing observed total or grouped effectiveness; assume effectiveness is survival if Eff is not provided. |
| Part.times | vector containing end time points of each time interval, must be monotonically increasing, required if using PT method without Eff provided, also required to truncate grouped costs and effectiveness within time limit L if the cost/effect history is provided. |
| Z | vector, matrix, or dataframe containing covariates for net benefit regression; if not provided, will do unadjusted analysis using simple regression. |
| PS.Z | vector, matrix, or dataframe containing covariates matrix for propensity score model using logistic regression, used in doubly robust method, if not provided, will fit an unadjusted logistic regression (e.g., for randomized studies). |
| interaction | vector containing covariate names to be included in interactions with treatment, must be a subset of variable names in Z, otherwise will be ignored. |
| Method | method for estimation. 'SW' - simple weighted, 'PT' - partitioned, 'CC' - naive complete case, 'AL' - naive all data, doubly robust method requires either 'SW' or 'PT'. |
| Sep.K | logical, if TRUE, estimate K (survival function of censoring time, used in inverse probability of censoring weighting) using Kaplan-Meier estimator within each treatment separately, default=TRUE. |
| PS.trim | value between (0, 0.5) to trim extreme propensity scores outside the range of (PS.trim, 1-PS.trim), used in doubly robust method, default=0.1. Although big PS.trim is allowed (e.g., 0.4), using big value is not recommended in practice. |
| Doubly.Robust | logical, if TRUE, perform doubly robust method, default=FALSE. |
| Eff.only | logical, if TRUE, fit a regression with dependent variable as effect, default=FALSE. |
| Cost.only | logical, if TRUE, fit a regression with dependent variable as cost, default=FALSE. |
| lambda | vector or scalar containing cost-effectiveness threshold values. |
| L | time limit horizon, used to truncate event time, costs and effectiveness if they are outside this time limit, assuming cost and effectiveness are evenly spread within each time interval in truncation. Must be smaller than the longest follow-up time. |

**Value (Output saved in the fitted NetBenReg object)**

The fitted NetBenReg object is a list, each for a value of cost-effectiveness threshold lambda. For example, if the fitted object is fit, then fit[[1]] is a list containing results for the 1st value of lambda. Similarly, fit[[2]] is a list containing results for the 2nd value of lambda. If Eff.only=TRUE and Cost.only=TRUE, the last 2 elements are for effectiveness-only and cost-only regressions, respectively. The following describes the components saved in each element (e.g., fit[[1]]) for each lambda value.

| Method | method for estimation. |
| --- | --- |
| lambda | value of cost-effectiveness threshold. |
| Reg.type | type of the regression model (NBR, Effect, or Cost). |
| est | vector or scalar containing estimates for coefficients or causal average INB. |
| se | vector or scalar containing standard error estimates for coefficients or causal average INB. |
| covariance | covariance matrix for coefficient estimates in net benefit regression for non-doubly robust methods. |
| coef.table | dataframe for the table of coefficients or causal average INB (estimate, standard error, Wald test statistic, and p-value). |
| CEAC | CEAC value for the given lambda value, provided for doubly robust method and net benefit regressions without interaction, but not provided for net benefit regressions with interaction due to heterogeneous cost-effectiveness across subgroups. |
| int.name | vector containing covariate names in the interactions, only provided for non-doubly robust method. |
| covar1st | an example dataframe containing covariates for the 1st patient, only provided for non-doubly robust method. |
| Regmodel | coefficient table for the part of net benefit regression, only provided for doubly robust method. |
| PSmodel | coefficient table for the part of propensity score model using logistic regression, only provided for doubly robust method. |
| PS | estimated propensity scores, only provided for doubly robust method. |
| group | vector containing treatment group indicator, only provided for doubly robust method. |

**Examples**

Preparation.

Before using for the first time the NetBenReg package in R, the NetBenReg package needs to be installed. This can be done using the devtools package:

> devtools::install_github("shuaichencode/NetBenReg")

Load the NetBenReg package, data, and select cost-effectiveness threshold values:

> library(NetBenReg)

> data(CEdata)

> lambda=seq(0,6,0.5)

The function NetBenReg works with both continuous and categorical covariates, but categorical covariates need to be changed to factor in advance, which is already done in the data CEdata using the as.factor function.

Fit a covariate-adjusted net benefit regression without interaction using SW method with history of costs and effectiveness of QALY.

> fit1<-NetBenReg(Followup=CEdata$survival, delta=CEdata$dead,

group=CEdata$Trt, Cost=CEdata[,8:22], Eff=CEdata[,24:38],

Part.times=1:15, Method='SW', Z=CEdata[,5:7], Eff.only=TRUE,

Cost.only=TRUE, lambda=lambda, L=10)

> print(fit1)

If the Z= option is not included, unadjusted net benefit regressions will be fitted. Although detailed results are saved in fit1 in R, important results are printed out for each lambda value. The coefficient estimate for group is interpreted as the estimated covariate-adjusted INBs for a given lambda (or covariate-adjusted extra mean QALY/costs for effectiveness-/cost-only regressions) (bold in following output). The output is:

All n = 2000 , Used n = 2000

Time limit horizon L = 10

Censoring rate within L = 48.5 %

Method: Simple Weighted

lambda = 0:

Estimate Std.err Wald p

(Intercept) -18.11894523 0.3250253 3.107646e+03 0.000000e+00

**group -2.54654804 0.3901034 4.261321e+01 6.670731e-11**

Age651 -0.09676961 0.4146777 5.445737e-02 8.154811e-01

LBBB1 3.22440371 0.4175194 5.964107e+01 1.132427e-14

Female1 -0.40563647 0.3771137 1.156990e+00 2.820907e-01

<snip>

for Effect:

Estimate Std.err Wald p

(Intercept) 2.9483057 0.1302542 512.343915 0.000000e+00

**group 0.9482411 0.1462802 42.021024 9.029733e-11**

Age651 -0.5796059 0.1465873 15.634098 7.685613e-05

LBBB1 1.1995345 0.1460079 67.495209 2.220446e-16

Female1 0.1384287 0.1377967 1.009194 3.150960e-01

for Cost:

Estimate Std.err Wald p

(Intercept) 18.11894523 0.3250253 3.107646e+03 0.000000e+00

**group 2.54654804 0.3901034 4.261321e+01 6.670731e-11**

Age651 0.09676961 0.4146777 5.445737e-02 8.154811e-01

LBBB1 -3.22440371 0.4175194 5.964107e+01 1.132427e-14

Female1 0.40563647 0.3771137 1.156990e+00 2.820907e-01

From the results, we can see that cost-only regression is equivalent to setting the cost-effectiveness threshold lambda=0 and then switching the signs of all coefficient estimates. There is “1” after the covariate name (e.g., LBBB1) which means that LBBB=0 is the reference group so here the coefficient estimate is for LBBB=1. The reference group can be redefined using relevel function if needed.

If there are missing values (e.g., in covariates, treatment, or follow-up time), the patients with missing values will be excluded, and hence the Used n = 2000 in the output will decrease due to discarding observations. We could also use other imputation packages together with NetBenReg (e.g., mice R package to perform multiple imputation) to handle the missing data.

To quickly calculate the adjusted ICER, we can simply fit cost-only and effect-only regressions without providing lambda (or let lambda=NULL):

fit1_0<-NetBenReg(Followup=CEdata$survival, delta=CEdata$dead,

group=CEdata$Trt, Cost=CEdata[,8:22], Eff=CEdata[,24:38],

Part.times=1:15, Method='SW', Z=CEdata[,5:7],

Eff.only=TRUE, Cost.only=TRUE, **lambda=NULL**, L=10)

Fit an unadjusted net benefit regression.

Covariates are not necessary sometimes (e.g., for randomized studies), and we can fit the unadjusted regressions without covariates by removing the option Z.

fit1_1<-NetBenReg(Followup=CEdata$survival, delta=CEdata$dead,

group=CEdata$Trt, Cost=CEdata[,8:22], Eff=CEdata[,24:38],

Part.times=1:15, Method='SW', Eff.only=TRUE, Cost.only=TRUE,

L=10, lambda=lambda)

Fit a covariate-adjusted net benefit regression without interaction using SW method using total cost and effectiveness only.

If only a vector of total costs and a vector of total effectiveness are available, Part.times is not required. However, if cost and effectiveness history is available, providing history data may help better truncate them within *L*. The following code will simply prorate the total 15-year costs (tot.cost) and 15-year effectiveness (tot.QALY) into *L*=10 years in analysis (total cost and effectiveness are bold in following code):

> fit2_1<-NetBenReg(Followup=CEdata$survival, delta=CEdata$dead,

group=CEdata$Trt, **Cost=CEdata$tot.cost, Eff=CEdata$tot.QALY**,

Method='SW', Z=CEdata[,5:7], Eff.only=TRUE, lambda=lambda,

L=10)

For example, if a patient was followed over 14 years with total observed costs of $25,000, the NetBenReg function will calculate the 10-year observed costs by $25,000/14$\times$10=$17,857, assuming costs were spread evenly over time (since cost history is not provided). This proration is not accurate since cost accumulation is often not evenly occurring through time. Two options can improve the estimation for this example, which lead to the same results:

(1) Provide yearly cost (and effectiveness) history so that NetBenReg function can calculate 10-year costs:

> fit2_2<-NetBenReg(Followup=CEdata$survival, delta=CEdata$dead,

group=CEdata$Trt, **Cost=CEdata[,8:22], Eff=CEdata[,24:38]**,

**Part.times=1:15,** Method='SW', Z=CEdata[,5:7], Eff.only=TRUE,

lambda=lambda, L=10)

(2) Calculate the total observed costs (and effectiveness) within 10 years before fitting the models and then provide them for NetBenReg function. Note that, if patients’ follow-up times are longer than 10 years, one must manually re-define their follow-up times to be limited to 10 years (i.e., the patients have complete 10-year data):

> CEdata$tot.cost10=apply(CEdata[,8:17],1,sum)

> CEdata$tot.QALY10=apply(CEdata[,24:33],1,sum)

> CEdata$survival10=pmin(CEdata$survival,10)

> fit2_3<-NetBenReg(**Followup=CEdata$survival10,** delta=CEdata$dead,

group=CEdata$Trt, **Cost=CEdata$tot.cost10,**

**Eff=CEdata$tot.QALY10**, Method='SW', Z=CEdata[,5:7],

Eff.only=TRUE, lambda=lambda, L=10)

The first two lines calculate the total observed costs (and effectiveness) within 10 years. The 3^rd^ line calculates new restricted 10-year follow-up time. This leads to the same results as the option (1).

Fit a covariate-adjusted net benefit regression without interaction using SW method using life years (LY) as effectiveness.

We can also fit a net benefit regression using life years as effectiveness, for which the option Eff is not needed, and the follow-up time will be used to calculate effectiveness directly:

> fit2_4<-NetBenReg(Followup=CEdata$survival, delta=CEdata$dead,

group=CEdata$Trt, Cost = CEdata[,8:22], **Eff=NULL**,

Part.times=1:15, Method='SW', Z=CEdata[,5:7],

Eff.only = TRUE, lambda=lambda, L=10)

Fit a covariate-adjusted net benefit regression without interaction using PT method with history of costs and effectiveness of QALY.

> fit2_5<-NetBenReg(Followup=CEdata$survival, delta=CEdata$dead,

group=CEdata$Trt, Cost=CEdata[,8:22], Eff=CEdata[,24:38],

Part.times=1:15, **Method='PT'**, Z=CEdata[,5:7], Eff.only=TRUE,

lambda=lambda, L=10)

Fit a net benefit regression for dataset with unequal time intervals.

The above program analyzed data with equal time intervals (e.g., yearly cost and QALY), which means that the time intervals are [0,1], (1,2], (2,3], (3,4], ..., (14,15] for cost and QALY histories in the dataset. However, the NetBenReg function also works for dataset with unequal time intervals. For illustration, we pretend that the time intervals in dataset do not have the same length (although not true for this dataset). Assume cost.1 (and QALY.1) is the cost (and QALY) accumulated in the first 2-year interval, cost.2 (and QALY.2) and cost.3 (and QALY.3) are cost (and QALY) accumulated in the following two 6-month intervals. Other time intervals keep the same. Thus, the time intervals are [0,2], (2,2.5], (2.5,3], (3,4], ..., (14,15] for cost and QALY histories, which can be introduced to NetBenReg by setting Part.times. Here is example code for such dataset with unequal time intervals:

> fit2_unequal<-NetBenReg(Followup=CEdata$survival,delta=CEdata$dead,

group=CEdata$Trt,Cost=CEdata[,8:22],Eff=CEdata[,24:38],

**Part.times=c(2,2.5,3:15)**, Method='PT', Z=CEdata[,5:7],

Eff.only=TRUE, lambda=lambda, L=10)

Possible issue in variable name when only one covariate is provided.

When using one covariate as a vector for option Z, if the name is not correct, R may show the default name Z. The following code provides the covariate stored in the 6^th^ column in data without its name:

> fit2_6<-NetBenReg(Followup=CEdata$survival, delta=CEdata$dead,

group=CEdata$Trt, Cost=CEdata[,8:22], Eff=CEdata[,24:38],

Part.times=1:15, Method='PT', **Z=CEdata[,5]**,

Eff.only=TRUE, lambda=lambda, L=10)

> print(fit2_6)

lambda = 0:

Estimate Std.err Wald p

(Intercept) -17.4835554 0.2667715 4295.172294 0.000000e+00

group -1.3430141 0.3418485 15.434540 8.541279e-05

**Z1** 0.4236033 0.3733751 1.287146 2.565746e-01

<snip>

To fix this issue, we may re-assign the name:

> fit2_7<-NetBenReg(Followup=CEdata$survival, delta=CEdata$dead,

group=CEdata$Trt, Cost=CEdata[,8:22], Eff=CEdata[,24:38],

Part.times=1:15, Method='PT', **Z=data.frame(Age65=CEdata[,5])**,

Eff.only=TRUE, lambda=lambda,L=10)

> print(fit2_7)

lambda = 0:

Estimate Std.err Wald p

(Intercept) -17.4835554 0.2667715 4295.172294 0.000000e+00

group -1.3430141 0.3418485 15.434540 8.541279e-05

**Age651**  0.4236033 0.3733751 1.287146 2.565746e-01

<snip>

Alternatively, we can use drop=FALSE option to prevent R from dropping the name:

> fit2_8<-NetBenReg(Followup=CEdata$survival, delta=CEdata$dead,

group=CEdata$Trt, Cost=CEdata[,8:22], Eff=CEdata[,24:38],

Part.times=1:15, Method='PT', **Z=CEdata[,5,drop=FALSE]**,

Eff.only=TRUE, lambda=lambda, L=10)

Fit a covariate-adjusted net benefit regression with interaction using PT method with history of costs and effectiveness of QALY.

> fit2<-NetBenReg(Followup=CEdata$survival, delta=CEdata$dead,

group=CEdata$Trt, Cost=CEdata[,8:22], Eff=CEdata[,24:38],

Part.times=1:15, Method='PT', Z=CEdata[,5:7],

**interaction=c("LBBB")**, Eff.only=TRUE, lambda=lambda, L=10)

> print(fit2)

lambda = 0:

Estimate Std.err Wald p

(Intercept) -17.8853239 0.3254077 3020.911785 0.000000e+00

**group -3.2362059 0.5033860 41.330437 1.285516e-10**

Age651 -0.1705199 0.3892581 0.191900 6.613399e-01

LBBB1 2.4484747 0.5599085 19.123051 1.225557e-05

Female1 -0.3688707 0.3502176 1.109360 2.922212e-01

**group:LBBB1 1.5203064 0.7239393 4.410197 3.572472e-02**

<snip>

for Effect:

Estimate Std.err Wald p

(Intercept) 3.1549636 0.1046052 909.666296 0.000000e+00

group 0.2702074 0.1612147 2.809216 9.372418e-02

Age651 -0.4470651 0.1139687 15.387580 8.756194e-05

LBBB1 0.3520552 0.1660203 4.496753 3.395929e-02

Female1 0.1143624 0.1069441 1.143542 2.849050e-01

group:LBBB1 1.4725388 0.2282211 41.631476 1.102045e-10

The option interaction= specifies the names of covariates which further have interactions with treatment, where the variable names must be a subset of those provided through the option Z. More than one interaction can be included, for example, interaction = c("Age65", "LBBB") to include two interactions with treatment, or interaction = names(CEdata[,5:7]) to include all three possible interactions with treatment.

Perform doubly robust method combining covariate-adjusted net benefit regressions with interaction and propensity scores.

> fit3<-NetBenReg(Followup=CEdata$survival, delta=CEdata$dead,

group=CEdata$Trt, Cost=CEdata[,8:22], Eff=CEdata[,24:38],

Part.times=1:15, Method='PT', Z=CEdata[,5:7],

interaction=c("LBBB"), **PS.Z=CEdata[,5:7], Doubly.Robust=TRUE**,

Eff.only=TRUE, lambda=lambda, L=10)

> print(fit3)

If the PS.Z= option is not specified, unadjusted logistic regression will be fitted, which assumes that all patients receive the new treatment with the same probability (such as a randomized study with a treatment assignment ratio of 1:2). Output:

All n = 2000 , Used n = 2000

Time limit horizon L = 10

Censoring rate within L = 48.5 %

Method: Doubly Robust Partitioned (estimate is causal average INB with given lambda)

lambda = 0:

Estimate Std.err Wald p

group -2.425465 0.3736994 42.1255 8.559953e-11

<snip>

for Effect:

Estimate Std.err Wald p

group 1.029909 0.1176782 76.59588 0

Details of the fitted propensity score model part and the net benefit regression part are also saved in fit3. For example, the following code prints out the saved results for the 1st value of lambda (i.e., = 0) for causal average INB, the net benefit regression part, and the propensity score part, respectively. Similarly, fit3[[2]] stores the results for the 2nd value of lambda.

> fit3[[1]]$lambda

[1] 0

> fit3[[1]]$Reg.type

[1] "NBR"

> fit3[[1]]$coef.table

Estimate Std.err Wald p

group -2.425465 0.3736994 42.1255 8.559953e-11

> fit3[[1]]$Regmodel

Estimate Std.err Estimate.1 p

(Intercept) -17.8853239 0.3254077 3020.911785 0.000000e+00

group -3.2362059 0.5033860 41.330437 1.285516e-10

Age651 -0.1705199 0.3892581 0.191900 6.613399e-01

LBBB1 2.4484747 0.5599085 19.123051 1.225557e-05

Female1 -0.3688707 0.3502176 1.109360 2.922212e-01

group:LBBB1 1.5203064 0.7239393 4.410197 3.572472e-02

> fit3[[1]]$PSmodel

Estimate Std. Error z value Pr(>|z|)

(Intercept) -0.48458949 0.08068323 -6.0060746 1.900687e-09

Age651 -0.66068101 0.10887725 -6.0681271 1.294105e-09

LBBB1 1.66027659 0.10260678 16.1809641 6.871008e-59

Female1 -0.05843057 0.10005462 -0.5839867 5.592292e-01

Construct CEAC plot based on the fitted models.

The following R code creates four CEACs (Figure 2 in the main text) based on the fitted net benefit regression models:

> plot(**fit2_5**,ylab="Probability new treatment is cost-effective",

xlab="Cost-effectiveness threshold (in $1000/QALY)", lwd=2,

pch=19, cex=1.2)

> plot(**fit2, subgroup=list(LBBB=0),** **add=TRUE**, col="gray50", lwd=2,

lty=2, pch=15, cex=1.2)

> plot(**fit2, subgroup=list(LBBB=1), add=TRUE**, col="gray50", lwd=2,

lty=3, pch=17,cex=1.2)

> plot(**fit3**, **add=TRUE**, lty=4, col="gray70", lwd=2, pch=0, cex=1.2)

> legend('right', c("Adjusted", "Subgroup:non-LBBB", "Subgroup:LBBB",

"Doubly Robust"), lty=c(1,2,3,4), lwd=c(2,2,2,2),

col=c(1, "gray50", "gray50", "gray70"), pch=c(19,15,17,0),

cex=c(1.2,1.2,1.2,1.2), seg.len =2.8)

The first line creates CEAC based on fit2_5, where the first option provides the fitted model from NetBenReg, and other options are parameters for plot to customize the curve. The 2nd and 3rd lines create CEACs for non-LBBB and LBBB subgroups (adjusted for age and gender), respectively, based on fit2, where the option add=TRUE adds this new curve to the existing plot instead of creating a new figure. Since fit2 is from a net benefit regression with interaction between LBBB and covariates, the option subgroup=list(LBBB=0) is required to specify the subgroup LBBB=0, where the subgroup is defined by the interaction term(s). If there are two interactions (e.g., interactions between treatment and LBBB and Age65) in the fitted model, we need to specify the values of both LBBB and Age65 to determine a subgroup, e.g., subgroup=list(LBBB=0, Age65=1). The 4th line adds the CEAC based on the doubly robust method (fit3), and the last line adds a legend. Details about other parameter options are in the help files of plot and legend for R.

**Examples with messages to help users identify issues**

The NetBenReg function provides user-friendly error or warning messages related to data issues, such as non-numerical costs and follow-up times, and inconsistent data dimensions. The following are error/warning examples that are frequently encountered by users.

Choose too large *L*.

The NetBenReg function will produce an error message if *L* is larger than the longest follow-up time. Although the NetBenReg function will produce estimates if we choose an *L* slightly smaller than longest follow-up time, the estimates might be unstable with large standard errors. Therefore, it is recommended to choose an *L* such that a “reasonable” number of subjects are still being observed at that time (e.g., choose *L* as the upper quartile of follow-up times).

> NetBenReg(Followup=CEdata$survival,

delta=CEdata$dead, group=CEdata$Trt, Cost=CEdata[,8:22],

Part.times=1:15, Method='SW', Z=CEdata[,5:7], Eff.only=TRUE,

lambda=lambda, **L=15**)

**Error** in NetBenReg(Followup = CEdata$survival, delta = CEdata$dead, group = CEdata$Trt, :

**Time limit L is greater than maximum of follow-up times. Choose a smaller L.**

*L* slightly smaller than the largest follow-up time.

The program will run with a warning. Note that standard errors are much larger with this large *L*, indicating unstable results.

> NetBenReg(Followup=CEdata$survival, delta=CEdata$dead,

group=CEdata$Trt, Cost=CEdata[,8:22], Part.times=1:15,

Method='SW', Z=CEdata[,5:7], Eff.only=TRUE, lambda=1, **L=14.5**)

lambda = 1:

Estimate Std.err Wald p

(Intercept) -11.4584242 1.153125 98.74089371 0.0000000000

group -0.3141828 1.235881 0.06462665 0.7993273650

Age651 -1.1987520 1.338378 0.80223456 0.3704261124

LBBB1 4.4923381 1.231987 13.29636192 0.0002659216

Female1 -1.5578020 1.229711 1.60479127 0.2052255367

for Effect:

Estimate Std.err Wald p

(Intercept) 8.9861565 0.8067029 124.0854176 0.00000000

group 1.5354586 0.7740708 3.9347293 0.04729875

Age651 -1.4723494 0.7850655 3.5173048 0.06073113

LBBB1 1.2612377 0.7186335 3.0802001 0.07925077

Female1 -0.6990202 0.7600967 0.8457496 0.35775753

**Warning** messages:

1: In NetBenReg_SW(X = FollowupL, delta = deltaL, Cost.total = Cost.total, :

**Estimate of probability of censoring < 10% at some time point. To have more stable results, could choose a smaller time limit L.**

Extremely small or large estimated propensity scores.

By default, the propensity scores are trimmed at 0.1 to prevent extreme values, that is, propensity scores smaller than 0.1 or larger than 0.9 will be trimmed to 0.1 or 0.9, respectively. However, it is easy to change the trimming value from 0.1 to other value, for example, use option PS.trim=0.05 in NetBenReg to change the trimming range to (0.05, 0.95). Extremely small or large estimated propensity scores (smaller than 0.1 or larger than 0.9) will lead to warnings when PS.trim is too small to trim them:

> CEdata1=CEdata

> set.seed(123)

> #let younger patients have a very high chance to be in Trt=1

> CEdata1$Trt[CEdata1$Age65==0]=rbinom(sum(CEdata1$Age65==0), 1,

prob=0.9)

> NetBenReg(Followup=CEdata1$survival, delta=CEdata1$dead,

group=CEdata1$Trt, Cost=CEdata1[,8:22], Eff=CEdata1[,24:38],

Part.times=1:15, Method='SW', Z=CEdata1[,5:7],

PS.Z=CEdata1[,5:7], Doubly.Robust=TRUE, **PS.trim=0.01**,

Eff.only=TRUE, Cost.only=TRUE, lambda=1, L=10)

<results omitted>

**Warning** messages:

1: In NetBenReg_SW_DR(X = FollowupL, delta = deltaL, Cost.total = Cost.total, :

**Maximum of estimated propensity score is 0.951. Recommend to use PS.trim to trim them.**

Extremely small or large estimated propensity scores with PS.trim to trim them will produce a message about the extreme value and trimming:

> NetBenReg(Followup=CEdata1$survival, delta=CEdata1$dead,

group=CEdata1$Trt, Cost=CEdata1[,8:22], Eff=CEdata1[,24:38],

Part.times=1:15, Method='SW', Z=CEdata1[,5:7],

PS.Z=CEdata1[,5:7], Doubly.Robust=TRUE, **PS.trim=0.1**,

Eff.only=TRUE, Cost.only=TRUE, lambda=1, L=10)

**Maximum of estimated propensity score is 0.951. PS are trimmed by 1-PS.trim=0.9.**

<results omitted>

Examine estimated propensity scores.

Additionally, the following code can examine the distribution and creates histograms of the estimated propensity scores (Supplementary Figure S2):

> summary(fit3[[1]]$PS)

Min. 1st Qu. Median Mean 3rd Qu. Max.

0.2308 0.3675 0.6122 0.5245 0.7535 0.7642

> # use ggplot2 package to create figure

> require(ggplot2)

> fit3[[1]]$group <- factor(fit3[[1]]$group, levels = c("0", "1"),

labels = c("Trt=0", "Trt=1")) #add labels for group

> ggplot(CEdata,aes(x=fit3[[1]]$PS))+geom_histogram(binwidth =0.1)+

facet_grid(rows = fit3[[1]]$group)+

xlab("Estimated propensity score")+ylab("Frequency")+

xlim(c(0,1))

Negative PS.trim for propensity score trimming.

Negative PS.trim will lead to a warning that propensity scores are not trimmed:

> NetBenReg(Followup=CEdata$survival, delta=CEdata$dead,

group=CEdata$Trt, Cost=CEdata[,8:22], Eff=CEdata[,24:38],

Part.times=1:15, Method='SW', Z=CEdata[,5:7],

Doubly.Robust=TRUE, PS.trim=-0.1, Eff.only=TRUE,

Cost.only=TRUE, lambda=1, L=10)

<snip>

**Warning** message:

In NetBenReg(Followup = CEdata$survival, delta = CEdata$dead, group = CEdata$Trt, :

**PS.trim is negative. Propensity scores are not trimmed.**

Negative costs or effectiveness.

QALY as effectiveness is allowed to be negative, since sometimes QOL could be negative (e.g., the nominal range of the EQ-5D index scores is 0 to 1, but negative EQ-5D index scores as low as -0.59 are possible for health states deemed to be worse than death). However, a warning will be provided for negative costs, although the function NetBenReg will still run:

> CEdata1=CEdata

> CEdata1[1,8]=CEdata1[1,24]=-1

> NetBenReg(Followup=CEdata1$survival, delta=CEdata1$dead,

group=CEdata1$Trt, Cost=CEdata1[,8:22], Eff=CEdata1[,24:38],

Part.times=1:15, Method='SW', Z=CEdata1[,5:7], Eff.only=TRUE,

Cost.only=TRUE, lambda=1, L=10)

<results omitted>

**Warning** message:

In NetBenReg(Followup = CEdata1$survival, delta = CEdata1$dead, :

**There is negative value in Cost.**
